# Photodynamic Evaluation of Synthesized Chlorin-Desthiobiotin Conjugate with Chemotherapeutic Drugs in Triple-Negative Breast Cancer Cells In Vitro and in Hydra Organisms In Vivo

**DOI:** 10.3390/ijms26115357

**Published:** 2025-06-03

**Authors:** Bailey N. Rutkowski, Meden F. Isaac-Lam

**Affiliations:** Department of Chemistry and Physics, Purdue University Northwest, 1401 S US Hwy 421, Westville, IN 46391, USA

**Keywords:** photodynamic therapy, PDT, triple-negative breast cancer, TNBC, chemotherapy, chemophotodynamic therapy, chlorin-vitamin conjugates, photosensitizers, paclitaxel, cisplatin, fluorouracil, hydra model organism

## Abstract

In this article, the synthesis and characterization of chlorin-based photosensitizers for potential applications in photodynamic therapy (PDT) of triple-negative breast cancer (TNBC) are described. The photodynamic efficacy of the synthesized chlorin-desthiobiotin (CDBTN) conjugate and its zinc and indium complexes were compared with the starting unconjugated precursor methyl pheophorbide, and assessed in a TNBC cell line in vitro. The chlorin-desthiobiotin complex aims to target the vitamin receptors upregulated in malignant cancer cells. The synthesized CDBTN was combined with chemotherapeutic agents (paclitaxel, cisplatin or fluorouracil) to evaluate their binary photodynamic efficacy. Cell survival assay in vitro indicated that the chlorin-vitamin conjugate CDBTN—alone and in combination with paclitaxel or fluorouracil—is photoactive against the TNBC cell line, but not when combined with cisplatin. The combination index (CI) calculated using the Chou-Talalay method indicated synergism of CDBTN and fluorouracil combination, aligning with the in vitro assay. The photodynamic cytotoxicity of CDBTN was also evaluated in vivo using the hydra as a novel model organism. This study is the first to show the use of the aquatic hydra organism in assessing photodynamic activity of the photosensitizer alone or in combination with chemotherapeutic agents. In vivo results with hydras indicated that the CDBTN-cisplatin combination is more phototoxic than CDBTN-paclitaxel or CDBTN-fluorouracil binary treatment. With the proper adjustment of concentration and light dosage, the synthesized photosensitizer can provide promising application in binary chemotherapy PDT treatment of TNBC.

## 1. Introduction

### 1.1. Photodynamic Chemotherapy for Triple-Negative Breast Cancer

Photodynamic therapy (PDT) or phototherapy is a cancer treatment that requires a combination of light of specific wavelength with a light-activated chemical entity known as a photosensitizer (PS) that reacts with endogenous molecular oxygen generating reactive oxygen species (ROS) in target tissues to elicit tumor cell destruction via oxidative damage [1,2,3]. PDT production of ROS causes oxidative stress affecting tumor blood vessels leading to vasoconstriction, endothelial cellular damage, thrombi formation, tumor microenvironment severe hypoxia, necrosis, and intratumor blood flow stagnation, which can also induce immunogenic cell death and systemic immune response [4]. Additionally, PDT can also promote the secretion of a series of cytokines to improve antigen presentation, recruitment, and infiltration of neutrophils and macrophages [5].

PDT, which is an effective and versatile treatment with a wide range of clinical applications, can be repeated multiple times, if necessary, and is crucial for managing recurrent conditions. The photosensitizing agent can be administered through a vein or applied topically to the skin. The advantages of PDT include normal tissue preservation, relatively non-invasive with less pain and bleeding tendency, and its ability to selectively treat tumor tissues using visible light under spatiotemporal control. PDT, approved in many countries (United States, Great Britain, France, Germany, and Japan), has been used for curative and palliative management of esophageal, bronchial, lung, gastric, cervical, skin, head, and neck cancers.

PDT may provide an alternative treatment for patients who have not been successful with standard breast cancer care management, specifically for triple-negative breast cancer (TNBC). TNBC is a type of breast cancer that shows low levels of estrogen receptor (ER), progesterone receptor (PR) and human epidermal growth factor receptor 2 (HER2) overexpression and/or gene amplification [6]. TNBC is characterized by an aggressive clinical behavior, higher rates of relapse, shorter overall survival, and poor outcome due to the lack of recognized molecular therapeutic targets. Chemotherapy involving platinum salts (cisplatin and carboplatin), taxane and anthracycline-based regimens remains the standard therapeutic approach for all stages of TNBC [7,8]. Chemotherapeutic drugs travel all throughout the body affecting normal healthy cells causing side effects such as fatigue, nausea, vomiting, hair loss, bruising, bleeding, infection, constipation, and diarrhea, among others [9,10].

Major drawbacks of PDT thwarting its widespread therapeutic clinical application include localized treatment complicating destruction of deep-seated tumors, absorption of endogenous biomolecules hampering excited light penetration, therapeutic probes providing insufficient targeting performance in avoiding damage to normal tissues, and clinically-approved PSs being porphyrin-based with low bioavailability and protracted elimination half-lives [11].

Binary therapy utilizing a combination of chemotherapy and PDT may reduce the required drug dosage of chemotherapy, thereby alleviating its toxic side effects. In a continued effort to provide options for TNBC patients, research in PDT photosensitizers to improve efficacy is evidently of utmost interest. In our laboratory, we prepared a series of PSs linked to vitamins to specifically target vitamin receptors overexpressed in cancer cells [12]. In this study, the photodynamic efficacy of the combination of our synthetic chlorin-vitamin conjugate photosensitizer and chemotherapeutic drugs was evaluated in vitro in TNBC cells and in vivo using green hydra as the model organism.

### 1.2. Hydra as Model Organism

Hydras are multicellular organisms that belong to the phylum *Cnidaria* found in still or slow-moving freshwater in the northern temperate zone [13]. *Hydra viridissima* is known as the green hydra due to its coloration derived from the photosynthetic unicellular green algae *Chlorella vulgaris* which thrives symbiotically in its body. The hydra organism is typically 10 mm long, its body crowned with 8–10 tentacles which are approximately the same length as the body. Reproduction is either by budding or by gametes produced in multipotent stem cells. The method of reproduction for hydra is directly related to its food intake, and can be affected by the ambient temperature. Hydras are tubular, made up of two layers of epithelial muscular cells connected by a gelatinous extracellular matrix interspersed with highly elastic collagen fibers. The rest of the hydra is the body, referred to as the column, which consists of four parts: (1) the gastric section (between the tentacles and the first bud); (2) budding section (produces the buds); (3) peduncle (between the first bud and the basal disc); and (4) basal disc (foot-like structure used for attachment). The body also consists of two embryonic cell layers: the ectoderm and the endoderm (which lines the gastrointestinal water-filled cavity acting as the site of food digestion). The nervous system consists of a nerve net of neurons along the entire body column with a much higher nerve density observed at both extremities. The only opening to the sac-like digestive system is at the apex of the elevated cone from which a ring of tentacles emerges surrounding the mouth.

The tentacles are studded with nematocysts and battery cells that trap or paralyze small prey. In the laboratory, hydras are fed with freshly-hatched *Artemia nauplii* (brine shrimp). Hydra culture can be maintained easily by careful monitoring of the culture and avoiding fungal growth, which is a common contamination in older *nauplii*. A typical meal for a hydra can range from 1 *naplius* to 2–3 *nauplii*. Cleaning dishes and removing debris after feeding is key in the proper maintenance of healthy hydras [14].

Hydras are freshwater sedentary organisms typically attached to stones, pebbles or aquatic plants, but can be released from the substratum, and then swim. Even though hydra are sessile, they can move in short bursts. Hydras have a remarkable ability to regenerate amputated body parts, making them virtually immortal. Due to the simple tubular body of the hydra, its epithelial cells are in constant contact with the environment allowing the entire body surface to interact with toxic substances in the aquatic system. Hydras cannot tolerate polluted water and do not survive in impaired water systems. A recent orthologome study showed that the hydra shares 6071 genes with humans, in contrast to the fruit fly *Drosophila melanogaster* and the nematode worm *Caenorhabditis elegans*, which share only 5696 and 4571 genes, respectively, with humans [15]. Hence, the hydra organism has been utilized as a powerful model animal for aging studies [16] and for studying toxicological effects of chemical pollutants in the ecosystem [17].

## 2. Results

### 2.1. Chemical Synthesis of Photosensitizers

**Scheme 1:**
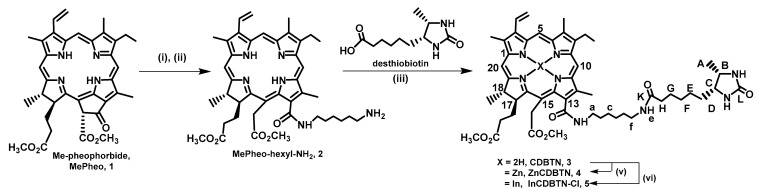
Synthesis of desthiobiotin-linked chlorin derivative and its corresponding zinc and indium complexes. Reaction conditions: (i) H_2_N(CH_2_)_6_NHBoc, CH_2_Cl_2_; (ii) trifluoroacetic acid (TFA), CH_2_Cl_2_; (iii) desthiobiotin, DMTMM; (iv) Zn(OAc)_2_ in MeOH; and, (v) InCl_3_ in NaOAc/HOAc.

Photosensitizers, also referred to as chromophores, composed of methyl pheophorbide 1 chemically linked to desthiobiotin to obtain chlorin-desthiobiotin conjugate (CDBTN 3) and its corresponding zinc and indium complexes were successfully synthesized as illustrated in Scheme 1. Chlorin-based photosensitizers have been used for PDT applications due to their absorption in the 660 nm region which falls into the therapeutic window of 600–800 nm for optimal light tissue penetration [18]. Nucleophilic attack on the carbonyl functional group at the 13′ position of methyl pheophorbide 1 with a hexyl diamine linker caused opening of the exocyclic ring, and forming the amine-functionalized pheophorbide 2 upon deprotection. Desthiobiotin, a biotin (Vit B7) analogue, was appended to the chlorin methyl pheophorbide via the hexyl diamine linker using a common peptide coupling procedure [19]. The six-carbon spacer connecting desthiobiotin to the chlorin ring was based on previous study by other investigators in which folic acid (Vit B9) was attached to a fluorophore. The six-carbon hexyl arm provided enough length for enhanced binding ability to the vitamin receptors or the sodium-dependent multivitamin transporter (SMVT) overexpressed on the cancer cell surface [20,21,22,23], and reduced the steric interaction caused by the bulky macrocyclic chlorin ring structure that can interfere with receptor binding [24].

Zinc 4 and indium 5 complexes were also prepared based on previous investigations demonstrating photodynamic effects of zinc and indium metallated PS. Zinc is essential for the activity of enzymes and transcription factors which control different fundamental biochemical pathways and cellular functions such as gene expression, DNA replication, DNA repair and apoptosis [25]. An earlier study reported the in vitro PDT activity of two morpholine-substituted Zn(II) metallated phthalocyanines conjugated to graphene quantum dots (GQDs) and biotinylated GQDs (GQDs-biotin) by noncovalent π-π interactions. Increased drug uptake and photoactivity was observed with Pcs–GQDs–biotin conjugates in human cancer cell lines [26]. Metallated phthalocyanine incorporated into a nanoscale organic framework was prepared and was shown to significantly enhance ROS generation upon light irradiation. With higher cellular uptake, enhanced ROS generation, and better biocompatibility, the zinc-complex nanostructure-mediated PDT exhibited an IC_50_ of 0.14 μM and achieved exceptional antitumor efficacy with >99% tumor growth inhibition and 80% cure rates on two murine colon cancer models [27]. Indium(III) complexes are versatile species with a broad range of biological applications including antibacterial, antifungal, antiviral and anticancer activities, and other applications in bioimaging, radiopharmaceutical, photodynamic chemotherapy and as antioxidants. In cancer therapy, indium compounds have been shown to enhance the apoptotic process in tumor cell destruction [28]. Previous in vitro and in vivo studies indicated that the indium complex of meso-tetraphenylporphyrin was about 1.5 times more effective in the photooxidation of red blood cells than Photofrin, an FDA-approved photosensitizer for PDT [29,30]. Thus, these metallated zinc– and indium–CDBTN complexes were prepared in this study.

Synthesized compounds were purified by chromatographic methods and were characterized by NMR (^1^H-, ^13^C-, COSY, HSQC) and mass spectrometric techniques. Sample purity was determined by reverse-phase high-performance liquid chromatography (RP-HPLC).

**Figure 1:**
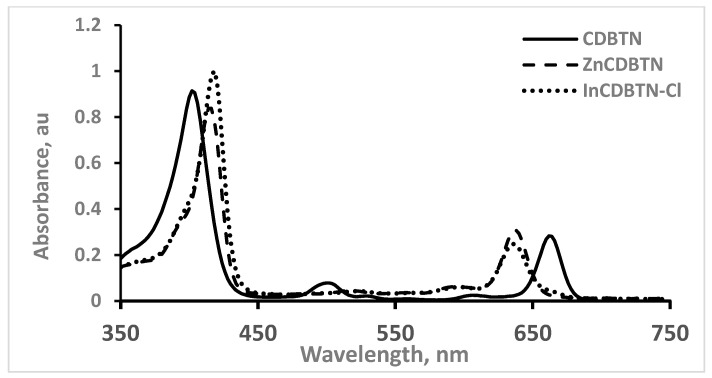
UV-vis absorption spectra in dichloromethane of chlorin-desthiobiotin conjugate (CDBTN) 3, ZnCDBTN 4, InCDBTN-Cl 5.

Figure 1 shows the UV-vis absorption spectra of the synthesized chlorin-desthiobiotin conjugate 3, and its corresponding zinc 4 and indium 5 complexes. Compared to unmetallated CDBTN, the zinc and indium complexes showed a 14–16 nm shift to longer wavelengths of the Soret band (from 402 nm to 416–418 nm) and 24–26 nm shift to shorter wavelengths of the Q band (from 662 nm to 636–638 nm). The typical bathochromic (red, longer) and hypsochromic (blue, shorter) shifts for the Soret and the fourth Q band, respectively, are generally observed for metallated porphyrin and chlorin complexes [31,32,33].

### 2.2. Binary Photodynamic Efficacy in Triple-Negative Breast Cancer Cells

**Figure 2:**
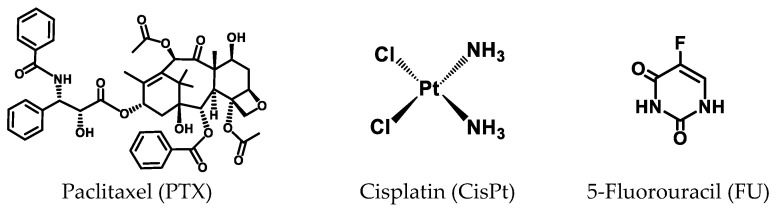
Molecular structures of chemotherapeutic agents.

The photosensitizers used in this study are methyl pheophorbide (MePheo) and our synthesized PSs derived from MePheo that include CDBTN with its zinc and indium complexes, namely, ZnCDBTN and InCDBTN-Cl, respectively. The chemotherapeutic drugs are paclitaxel (PTX), cisplatin (CisPt), and 5-fluorouracil (FU), shown in Figure 2.

**Figure 3:**
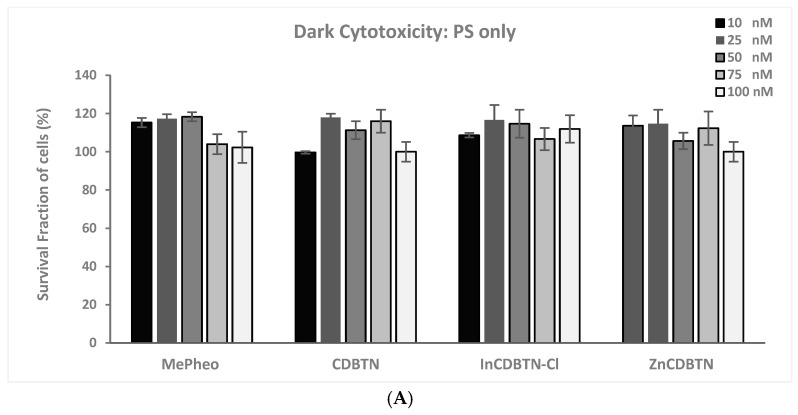

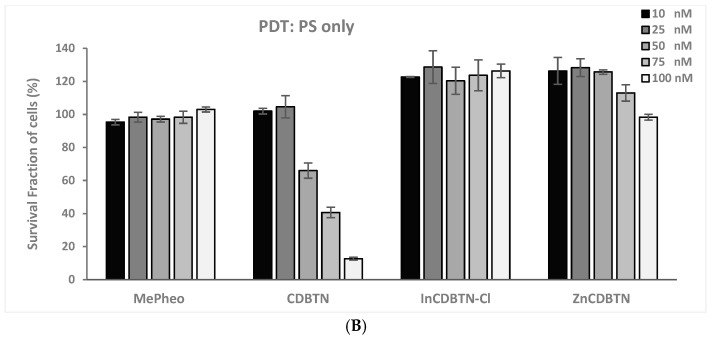
Bar plots of cell survival assay of triple-negative BT-549 breast cancer cell line after 24 h PS (MePheo, CDBTN, InCDBTN-Cl, and ZnCDBTN) treatment: (**A**) in the dark, and (**B**) after light exposure. Cells were treated with varying concentrations (10, 25, 50, 75, and 100 nM) of PSs. Light dose = 0.96 J cm^−2^. Data reported are the mean ± SD of triplicate measurements in three separate experiments.

The dark cytotoxicity and light-exposed PS-treated TNBC cells are shown in Figure 3A,B, which indicates that no dark toxicity of the PSs and only the unmetallated PS CDBTN exhibited a dose-dependent effect.

The starting precursor MePheo, serving as a control, and the metallated ZnCDBTN and InCDBTN-Cl did not cause photodynamic effects in the 10–100 nM concentration range used upon light exposure as indicated in Figure 3B. The zinc and indium complexes seemed to protect the cells from photooxidative damage. In our previous studies of zinc and indium complexes, zinc-chlorin complexes did not cause any significant photodynamic effect compared to indium-chlorin complexes. However, not all indium complexes produced the expected and desired tumor cell photocytotoxicity. In previous studies, our group created several indium metallated chlorophyll derivative conjugates containing vitamins or vitamin analogues that include indium complexes of chlorin-lipoic acid (InCLA-Cl), chlorin-pantothenic acid (InCPA-Cl), chlorin-biotin (In-CBTN-Cl), and chlorin-bexarotene (InCBX-Cl). From the in vitro studies conducted, only InCLA-Cl demonstrated significant photodynamic activity against TNBC and prostate cancer lines. InCLA-Cl at a concentration of 75 nM reduced the cell viability of BT-549 TNBC cell line by 64%, while at 200 nM, a 30% reduction of PC-3 prostate cancer line was observed. A light dose of 0.96 J cm^-2^ was used in both measurements [12,34]. Their corresponding unmetallated chlorin-vitamin conjugates are also observed to be more photoactive than the indium complexes. This observation seems to be contrary to what has been documented that metal coordination of indium to chlorin derivatives enhances higher singlet oxygen production [35]. It is conceivable that the electronic nature, polarity and steric effects of the attached vitamin such as pantothenic acid, biotin, desthiobiotin, and bexarotene affected the ability of indium to generate enough singlet oxygen to initiate the photodynamic efficacy. However, it is worth noting that our synthesized PS proved to be better than the starting compound MePheo as shown in the cell viability assay.

**Figure 4:**
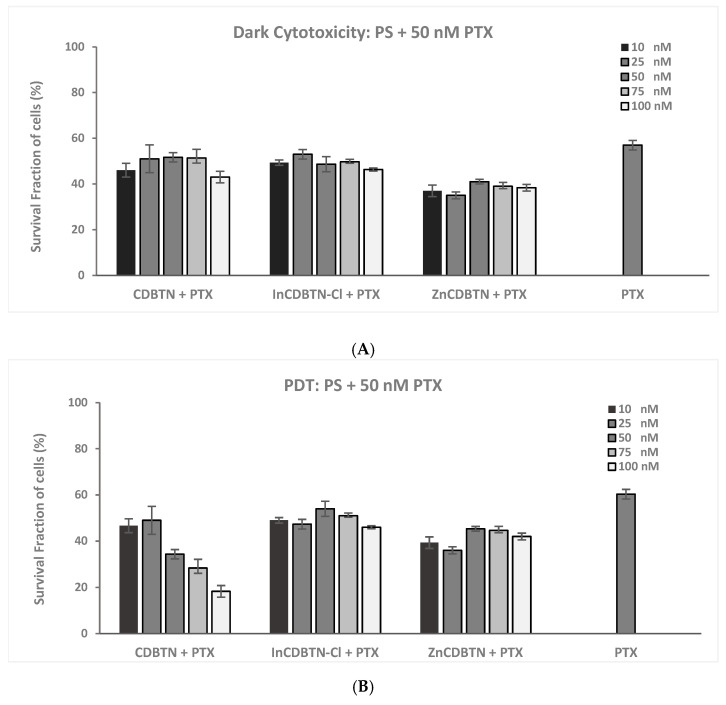
Bar plots of cell survival assay of triple-negative BT-549 breast cancer cell line after 24 h PS (CDBTN, InCDBTN-Cl, and ZnCDBTN) co-treated with PTX (**A**) in the dark, and (**B**) after light exposure. Cells were treated with varying concentrations (10, 25, 50, 75, and 100 nM) of PSs. PTX concentration is 50 nM. Light dose = 0.96 J cm^−2^. Data reported are the mean ± SD of triplicate measurements in three separate experiments (*p* < 0.05, two-tailed *t*-test).

Binary treatment using PTX and the synthesized PSs in the dark caused a reduced cell survival, averaging 40%, which is about 20% less than when cells were treated only with PTX at 50 nM (Figure 4A). Upon light treatment, dose dependence was observed only for the CDBTN–PTX combination, but not for the metallated CDBTN (Figure 4B).

**Figure 5:**
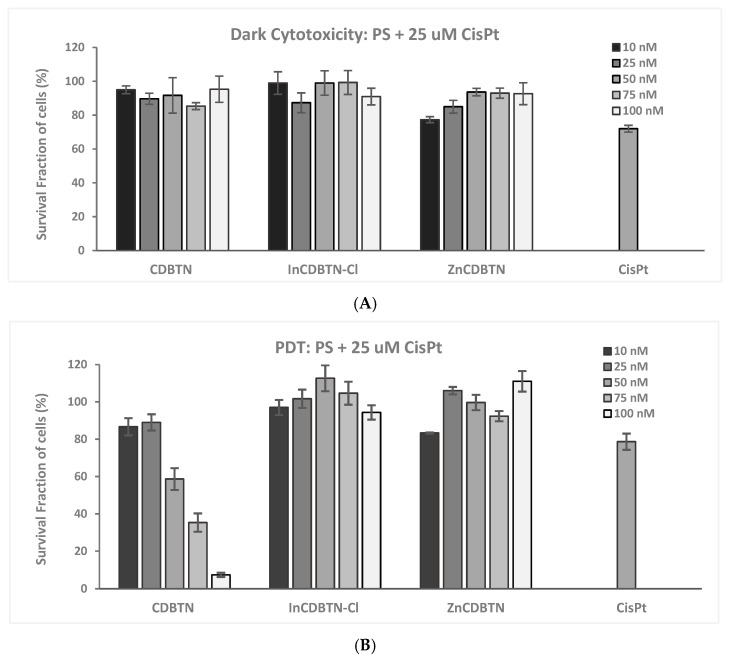
Bar plots of cell survival assay of triple-negative BT-549 breast cancer cell line after 24 h PS (CDBTN, InCDBTN-Cl, and ZnCDBTN) co-treated with CisPt (**A**) in the dark, and (**B**) after light exposure. Cells were treated with varying concentrations (10, 25, 50, 75, and 100 nM) of PSs. CisPt concentration was 25 μM. Light dose = 0.96 J cm^−2^. Data reported are the mean ± SD of triplicate measurements in three separate experiments. CDBTN at 50–75 nM showed a statistically insignificant effect upon combination with CisPt (*p* > 0.05, two-tailed *t*-test).

**Figure 6:**
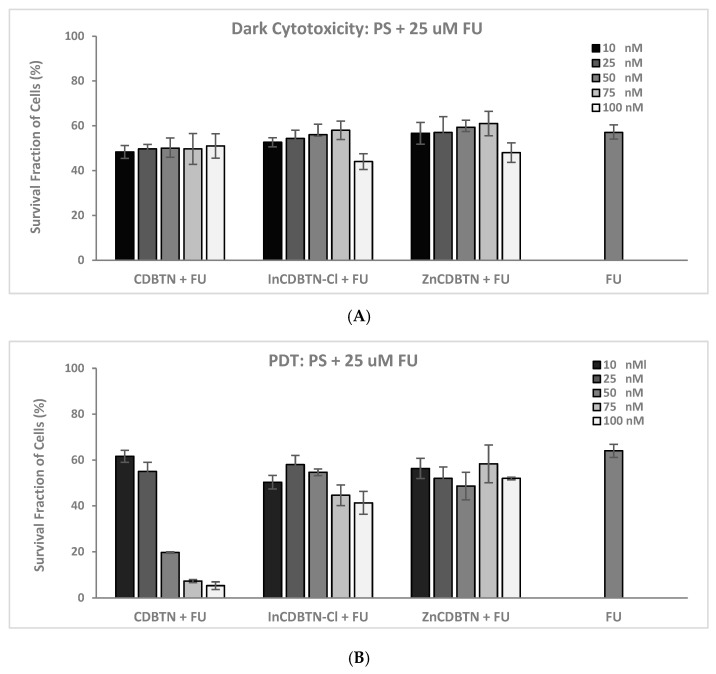
Bar plots of cell survival assay of triple-negative BT-549 breast cancer cell line after 24 h PS (CDBTN, InCDBTN-Cl, and ZnCDBTN) co-treated with FU (**A**) in the dark, and (**B**) after light exposure. Cells were treated with varying concentrations (10, 25, 50, 75, and 100 nM) of PSs. FU concentration was 25 μM. Light dose = 0.96 J cm^−2^. Data reported are the mean ± SD of triplicate measurements in three separate experiments (*p* < 0.05, two-tailed *t*-test).

Binary treatment using CisPt and the synthesized PSs in the dark did not decrease cell survival (Figure 5A). The effect of FU (Figure 6A) is more pronounced than for CisPt, in which cell survival for the combined CDBTN-FU treatment was the same as for FU alone.

Upon light treatment, dose dependence was observed only for CDBTN–CisPt as well as for the CDBTN–FU combination, which is similar to what was detected with CDBTN–PTX, but not for the metallated CDBTN (Figure 4B, Figure 5B and Figure 6B). The concentration used for CisPt and FU is 25 μM, while only 50 nM was used for PTX. Additionally, CisPt did not cause any substantial cell inhibition in the CDBTN–CisPt combination with light treatment. Compared to CDBTN alone, there is a slight decrease in cell survival for CDBTN–CisPt from 66% to 59% and 41% to 35% using a concentration of 50 nM and 75 nM CDBTN, respectively, resulting in a statistically insignificant (with *p* > 0.05, two-tailed *t*-test) inhibition. It is possible that the presence of platinum reduced the photodynamic efficacy of CDBTN and its indium and zinc complexes. PTX and FU do not contain any metal compared to CisPt, and their binary combination is more pronounced than with CisPt in vitro (with *p* < 0.05, two-tailed *t*-test) at the nanomolar concentration of CDBTN.

### 2.3. Cell Viability Summary and Combination Index

Cell viability inhibition in TNBC cells is based on the cell survival (MTT) assay of the binary therapy by the combination effect of PDT and chemotherapeutic agents. In this study, it should be noted that monotherapy alone—either PDT with the photosensitizers (with light) or chemotherapy with chemotherapeutic agents (with or without light)—resulted in less treatment efficacy than combination treatments.

Table 1 tabulates percentage inhibition of TNBC cells treated with our synthesized PS CDBTN at 50–100 nM by itself, and simultaneously treated with PTX (50 nM), CisPt (25 μM), or FU (25 μM). TNBC cells were then exposed to light after binary treatment for 24 h. The binary combination of CDBTN and FU (80–95%) seemed to have the most inhibitory effect compared to CDBTN and PTX (66–81%) or CDBTN and CisPt (41–93%).

The Chou–Talalay method, an open-source mathematical tool, was employed to determine the combination index (CI) in calculating the dose–effect dynamics, correlating single entity and multiple entities [36]. Interaction dynamics of multiple entities resulted in a combination index equation, and this computer simulation offered an automatic quantitative determination of Synergism (CI < 1), Additive (CI = 1), and Antagonism (CI > 1). The unified dynamics algorithm uses a minimum of dose-data points to fit the general mass-action law principle.

The CI values for CDBTN based on the best biological activity to inhibit the growth of TNBC cells in combination with known PTX, CisPt and FU used in this study are listed in Table 2 as determined using CompuSyn software Version 1.0. *The combination of CDBTN and FU showed synergism with the lowest CI value of 0.55*. Other combinations in the table range from synergistic to nearly additive to antagonistic, to slightly or moderately antagonistic, with CI values ranging from 0.59–1.37. The calculated CI values are based on 50–75 nM concentrations for CDBTN, 50 nM PTX, 25 μM CisPt, and 25 μM FU.

Based on the low nanomolar and micromolar dosage used for the binary therapy, the best chemophotodynamic combination treatment against TNBC in decreasing order is the following:

CDBTN + FU > CDBTN + PTX > CDBTN + CisPt

### 2.4. Fluorescence Microscopy

PDT can trigger apoptosis and/or necrosis as the mode of cell death mechanism. Morphological characteristics of apoptosis as identified under light microscopy can include cell shrinkage, chromatin condensation, nuclear fragmentation, cell membrane blebbing, loss of adhesion and cellular volume, and the formation of apoptotic bodies [37,38]. On the other hand, necrotic cell death is manifested by swelling of cellular membranes together with chromatin condensation and membrane breakage [39,40]. Necrosis is much more inflammatory than apoptosis. However, both apoptosis and necrosis have been observed in PDT-induced cancer treatment [41].

**Figure 7:**
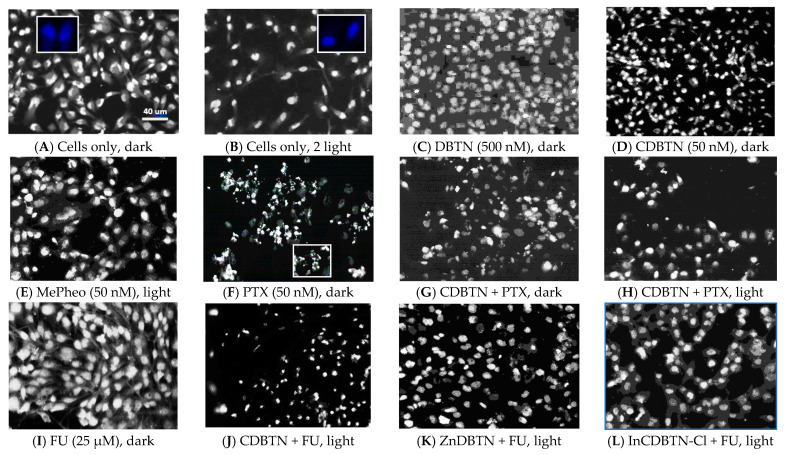
Fluorescence microscopy images of fixed human triple-negative breast cancer (TNBC) BT-549 cells. Grayscale and blue fluorescence images of cells stained with Hoechst 33258. Morphology of (**A**) untreated unirradiated cells (sham control); then, of (**B**) cells alone after treatment with 2 min light, and of cells after 24 h treatment with (**C**) DBTN, 500 nM; (**D**) CDBTN (50 nM), dark; (**E**) MePheo, followed by 1 min light irradiation; (**F**) PTX, 50 nM (dark); (**G**) CDBTN (50 nM) + PTX (50 nM) in the dark; (**H**) CDBTN (50 nM) + PTX (50 nM), 30 s light exposure; (**I**) FU, 25 μM (dark); and PSs at 50 nM combined with FU (25 μM), followed by 1 min light exposure: (**J**) CDBTN; (**K**) ZnCDBTN; and (**L**) InCDBTN-Cl, respectively. Apoptosis characterized by chromatin condensation and cell shrinkage is evident upon treatment with PTX (**F**) and with CDBTN with FU (**J**). Light exposure for 30 s and 1 min corresponds to light doses of 0.48 and 0.96 J cm^−2^, respectively. Scale bars are the same for all images as in the first figure. Objective used was 20X.

To determine the preferred mode of cell death pathway for the photodynamic chemotherapy in this study, a nuclear stain was employed to monitor cellular morphological alterations upon light exposure [42]. Fluorescence microscopy images of TNBC cells stained with Hoechst 33258 nuclear stain are displayed in Figure 7. Untreated and unirradiated TNBC cells are spindle-shaped with intact cytoplasm, diffused chromatin, well-defined cell membrane, and oval-shaped nuclei stained dark blue with the nuclear stain (Figure 7A,B). The same morphology was observed for untreated sham control and irradiated TNBC cells, indicating that that the light dosage used in this study did not cause any cellular changes. When treated for 24 h with 500 nM desthiobiotin (DBTN) and 50 nM CDBTN in the dark, no significant morphological alterations were observed (Figure 7C,D). Similarly, no changes were shown with the control sample of methyl pheophorbide (MePheo) at 24 h treatment followed by 1 min light exposure (Figure 7E). Figure 7F showed cells treated with 50 nM PTX in the dark with a clear manifestation of reduced cellular volume and nuclear density, and shrunk cells with chromatin fragmentation showing apoptosis as the preferred pathway for PTX-treated TNBC cells. The effect of PTX carried over in the presence of the synthesized PS CDBTN (Figure 7G,H) in the dark and with light exposure. The effect of blending PDT and chemotherapy is indicated when PTX was combined with CDBTN at 50 nM concentration and 30 s light irradiation. When FU was used at 25 μM, induction of cell destruction was ineffective in the dark (Figure 7I). Upon binary treatment with FU and PDT, only CDBTN indicated significant morphological changes including reduced nuclear and cellular density, but not with the metal complexes ZnCDTN nor InCDBTN-Cl (Figure 7J–L), confirming our cytotoxicity assay in Figure 6.

Fluorescence microscopy results in our study demonstrated that the apoptotic pathway seems to be the mode of cell death mechanism when PTX was used and when combined with PDT, and possibly also with FU treatment. Evidence of necrotic cell death was not apparent.

### 2.5. Transmission Electron Microscopy

**Figure 8:**
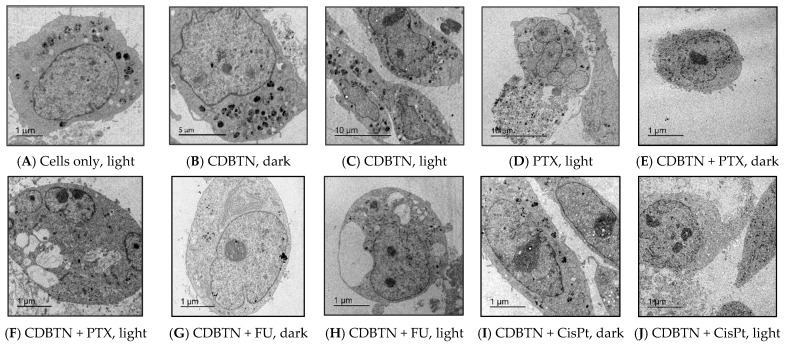
Ultrastructure TEM images of human triple-negative breast cancer (TNBC) BT-549 cells: (**A**) untreated irradiated for 1 min; (**B**) CDBTN-treated and unirradiated; (**C**) CDBTN-treated with 30 s light treatment; (**D**) PTX-treated treatment, with 30 s light treatment; (**E**) CDBTN-treated and co-treated with PTX without and (**F**) with 30 s light treatment; (**G**) with FU without, and (**H**) with 30 s light treatment; and, (**I**) with CisPt without and (**J**) 30 s light treatment. Irregularly shaped nuclear membrane is apparent in treated cells in the presence of light. Light dose in 30 s = 0.48 J cm^−2^, and in 1 min = 0.96 J cm^−2^. Concentrations: PS = 50 nM.

Other evidence of the mode of PDT-induced cell death mechanism can be characterized using the transmission electron microscopy (TEM) technique. TNBC cells with light exposure but without PS nor chemotherapeutic agent displayed normal cell size, shape and appearance with smooth cell membrane (Figure 8A). Upon 24 h treatment with CDBTN in the dark, the nuclear membrane started to become irregularly shaped (Figure 8B). When exposed to light for 30 s, CDBTN-treated and irradiated cells became distorted with loss of cellular integrity and some chromatin condensation (Figure 8C). TNBC cells treated with PTX for 24 h followed by irradiation for 30 s indicated fragmentation and compartmentalization of the nuclear envelope with numerous atypical vesicular components in the cytosol (Figure 8D). Co-treatment with CDBTN and PTX in the dark showed cell shrinking and darkened DNA condensation with loss of nuclear membrane integrity (Figure 8E). Upon irradiation, further DNA collapse together with vacuole formation is evident (Figure 8F). FU and CDBTN co-treated cells in the dark resulted in no drastic morphological changes except for some irregularity in the nuclear membrane (Figure 8G), but alterations in the appearance occurred in the presence of light in which large vacuoles and vesicles as well as nuclear fragmentation are obvious (Figure 8H). Similar results were observed when TNBC cells were co-treated with CDBTN and CisPt in the dark and during light exposure (Figure 8I,J).

The TEM images in our study seem to confirm our fluorescence microscopy results that apoptosis is the preferred mode of cell death during binary chemophotodynamic treatment. Due to the high resolution of the TEM technique, our TEM data suggest that only apoptosis occurs, but some autophagic cell death pathway is also happening [43]. Apoptosis, which is characterized by nuclear and cell shrinkage, and autophagy, which is identified as accumulation of cytosolic vacuoles and membranes, are suggested to occur when our synthesized PS was co-treated with PTX, FU or CisPt. Therefore, different cell death pathways happen simultaneously and are activated during photodynamic chemotherapy in this study.

### 2.6. Hydra as a Model Organism for In Vivo Study

**Figure 9:**
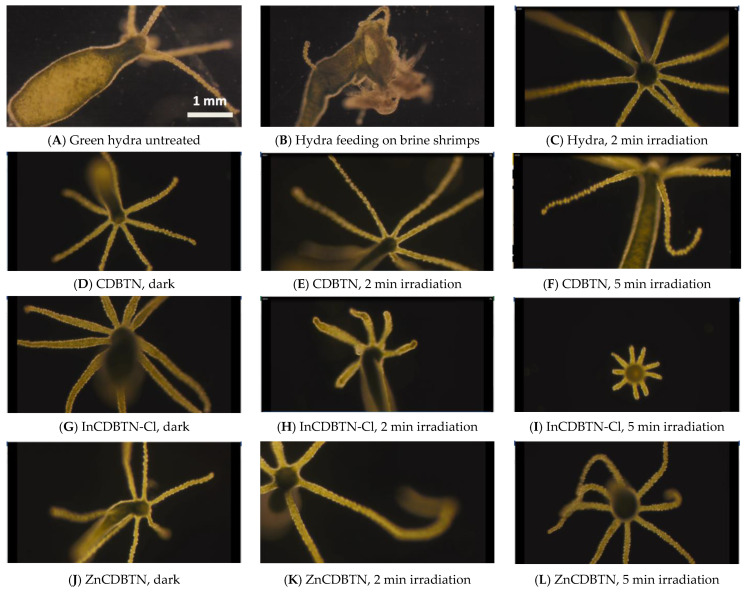
Morphological changes of hydra organisms treated with synthesized PSs: (**A**) Green hydra in the dark; (**B**) hydra being fed with brine shrimps; (**C**) hydra, irradiated for 2 min; (**D**–**F**) CDBTN, dark and irradiated; (**G**–**I**) InCDBTN-CL, dark and irradiated; (**J**–**L**) ZnCDBTN, dark and irradiated. Light dose in 2 min = 1.92 J cm^−2^ and 5 min = 4.8 J cm^−2^. Concentrations: PS = 1.0 μM. Scale bars are the same for all images as in the first figure. Objective used was 10X.

*Hydra viridissima* or the green hydra was utilized in our study as an in vivo model organism to determine the photodynamic efficacy of the synthesized CDBTN conjugate co-treated with PTX, CisPt or FU as the chemotherapeutic drugs. Morphology of hydras in the dark and during feeding with brine shrimps are shown in the images below (Figure 9A,B), and of the animal being irradiated for 2 min with its eight fully extended tentacles and no significant changes in its appearance (Figure 9C). Upon 24 h treatment with our synthesized CDBTN at a concentration of 1 μM in the dark and upon light exposure, hydra morphology indicated few changes (Figure 9D–F). Longer irradiation at 5 min (Figure 9F) caused its tentacles to not fully extend to some degree, but rather curved. Using InCDBTN-Cl also at 1 μM in the dark (Figure 9G–I) and in the presence of light showed drastic morphological changes that include slight swelling at the base of the tentacles, and a flowery phenotype as the extent of light exposure was prolonged from 2 min to 5 min. However, with the zinc complex ZnCDBTN in the same 1 μM concentration (Figure 9J–L), the effect on the morphology of the animal was less than with InCDBTN-Cl but more than the unmetallated CDBTN.

**Figure 10:**
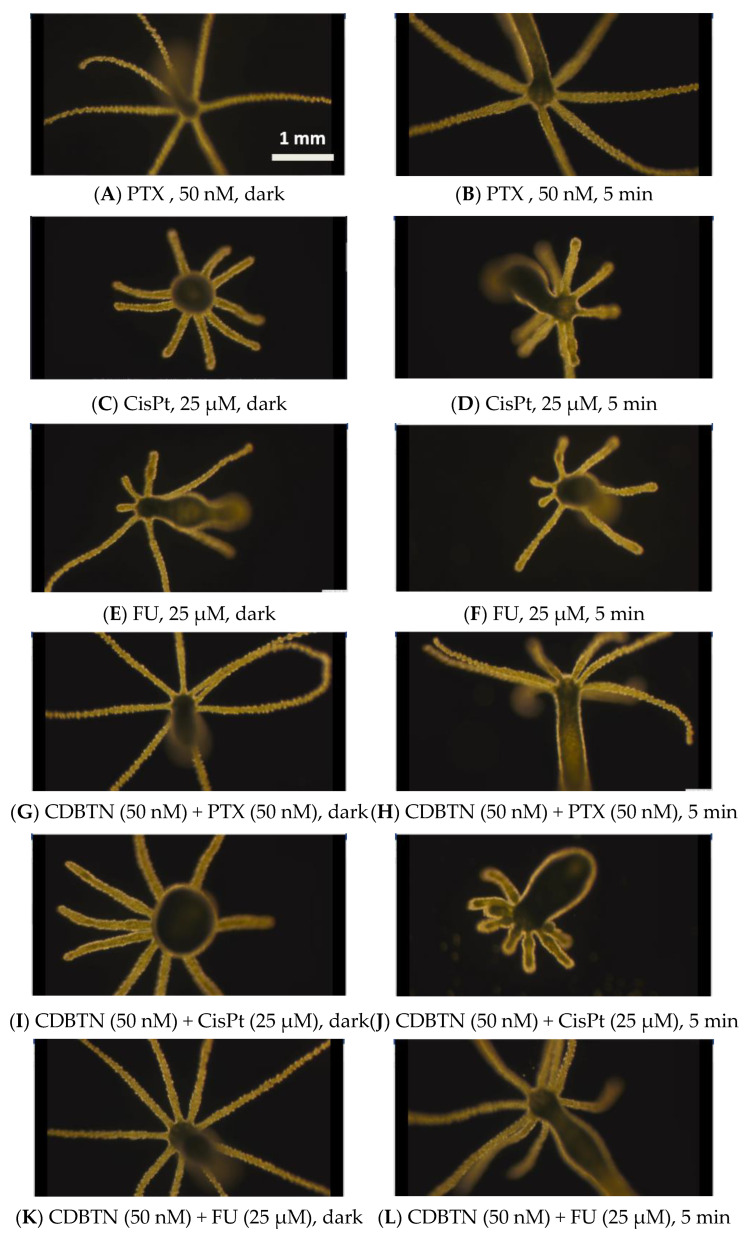
Morphological changes of hydra organisms treated with chemotherapeutic drugs and PS: (**A**) PTX in the dark; (**B**) PTX, irradiated; (**C**) CisPt in the dark; (**D**) CisPt, irradiated; (**E**) 5-FU in the dark; (**F**) FU, irradiated; (**G**) co-treatment with CDBTN and PTX in the dark; (**H**) co-treatment with CDBTN and PTX, irradiated; (**I**) co-treatment with CDBTN and CisPt in the dark; (**J**) co-treatment with CDBTN and CisPt, irradiated; (**K**) co-treatment with CDBTN and FU in the dark; and (**L**) co-treatment with CDBTN and PTX, irradiated. An irregularly shaped nuclear membrane is apparent in treated cells in the presence of light. Light dose in 5 min = 4.8 J cm^−2^. Concentrations: PTX = 50 nM; CisPt = 25 μM; FU = 25 μM; CDBTN = 50 nM. Scale bars are the same for all images as in the first figure. Objective used was 10X.

PTX, CisPt and FU were the chemotherapeutic drugs used in the in vitro study as described in the previous section. These were also used to evaluate their effects in combination with our synthesized PSs in vivo using the hydra as the animal model. The concentrations of chemotherapeutic drugs used in the hydra were the same concentrations used in the in vitro TNBC cells. PTX-treated hydras at 50 nM did not exhibit any changes as compared to CisPt- and FU-treated organisms at 25 μM, in which swelling and shortening of the tentacles were apparent (Figure 10A–F). Upon irradiation with a light dose of 4.8 J cm^−2^, only the combined CDBTN and CisPt-treated hydras produced the flowery phenotype which implied that a photodynamic effect played a role in causing a change. The combination of CDBTN with FU after light exposure seemed to have the opposite effect. The presence of CDBTN showed some type of protection from the effect of FU (Figure 10E,F), evident even after irradiation (Figure 10K,L).

The in vivo results with the hydra organism were not the same as the in vitro results in TNBC cells. Greater morphological changes in vivo with the hydra organism were observed for the metal complexes InCDBTN-Cl and ZnCDBTN than the unmetallated CDBTN upon light treatment. Furthermore, greater photodynamic effect was observed in vivo for the CDBTN–CisPt combination than the CDBTN–PTX and CDBTN–FU combinations in vitro. Our results suggested that CisPt caused the most damage to the hydra by itself, and with the combined treatment with our synthesized PS CDBTN. It is possible that the organism is more sensitive to the platinum metal in CisPt than the other chemotherapeutic drugs PTX and FU. Previous studies have demonstrated that metals such as copper and mercury are toxic to hydras, and thus the hydra has been utilized as a model organism to evaluate metal toxicity in the environment.

## 3. Discussion

### 3.1. In Vitro Chemophotodynamic Treatment

#### 3.1.1. PDT and PTX Combination

In this study, our synthesized photosensitizer CDBTN was demonstrated as a possible candidate for PDT–chemotherapy combination treatment for triple-negative breast cancer.

A previous study on combination treatment of porphyrin–lipid (18.3 wt%) with PTX (3.1 wt%) forming spherical core-shell nanoemulsions resulted in higher tumor accumulation in KB-tumor bearing mice and inhibited tumor growth by 78% in an additive manner, compared with PDT (44%) or chemotherapy (46%) alone [44]. The formulation was composed of 1,2-distearoyl-sn-glycero-3-phosphoethanolamine-N-[amino (polyethylene glycol)-2000], glyceryl trioctanonate together with varying amounts of PTX. The synthetic design created an amphiphilic self-assembly of a monodispersed nanoemulsion shell from a hydrophilic phosphate head group and a hydrophobic trioctanoate oily tail with an amphiphilic porphyrin–lipid functionality. This biocompatible and stable porphyrin–lipid nanoemulsion was designed with PTX loaded into the oil core to address the poor aqueous solubility of PTX, which hinders its effective circulation and tumor accumulation, and also facilitated PDT and chemotherapy combination treatment.

Another study was conducted on the application of combination therapy on pancreatic (PANC) cancer, an aggressive subtype of cancer with poor prognosis, known for its refractory nature [45]. A stable nanoplatform was established that combines a disulfide-bonded PTX-based prodrug which was mixed with gemcitabine and a photosensitizer THPP in an optimized ratio. Cell viability of PANC-1 cells treated with different conditions and combinations of PTX, GEM and THPP was significantly decreased, and reached the lowest level of 16.3 ± 0.5% specifically when treated with 10 μg/mL PTX and 650 nm laser (0.4 W/m^2^, 5 min). Both in vitro and in vivo experiments sufficiently demonstrated that the combination treatment led to a significant synergistic anti-tumor effect. However, this study did not provide further explanation as to the molecular basis of the synergism caused by the PDT–PTX–gemcitabine combination modality.

Our study appears to corroborate the results demonstrated by other investigations regarding the effect of PDT–PTX combination in that co-treatment of CDBTN with PTX produced a better photodynamic therapeutic outcome than PDT or PTX monotherapy alone. In vitro cell survival assay indicated dramatic reduction in TNBC cell proliferation by 52% for CDBTN-PTX co-treatment upon light exposure. CDBTN and PTX concentrations were 10 and 50 nM, respectively, at a light dose of 0.96 J cm^−2^. The therapeutic potential of PTX is based on its ability to bind to β-tubulin in microtubules enhancing α-tubulin acetylation and preventing depolymerization to trigger apoptosis in tumor cells [46]. PTX also promoted ROS generation by enhancing NADPH oxidase activity associated with plasma membranes, and increased extracellular O_2_ and H_2_O_2_ accumulation [47]. The level of α-tubulin acetylation of PTX-treated cells is reported to be further enhanced upon ROS exposure [48].

#### 3.1.2. PDT and CisPt Combination

A recent study was performed regarding the combination efficacy of CisPt-based chemotherapy and a photosensitizer (5-aminolevulenic acid or ALA) in TNBC cells [49]. That particular investigation indicated reduced cell viability following simultaneous treatment compared to sequential combination therapy. The simultaneous combination treatment of 2.5 µM CisPt and 5-ALA/PDT at 6 J/cm^2^ and 9 J/cm^2^ produced 46.78% and 53.6% apoptotic death, respectively, in TNBC cells in vitro compared with monotherapies (CisPt (37.88%) and 5-ALA/PDT (6 J/cm^2^: 31.48% and 9 J/cm^2^: 37.78%)). CisPt and 5-ALA/PDT combination treatment caused nuclear fragmentation and mitochondrial damage as morphological hallmarks of apoptosis. These results obtained by others suggest that CisPt and 5-ALA/PDT simultaneous combination therapy could be a promising new alternative strategy for treating TNBC.

Our results of CDBTN–CisPt combination seem to confirm the results obtained by others, in which binary treatment proved to be more effective than monotherapy alone.

#### 3.1.3. PDT and FU Combination

A previous study was conducted to investigate the effect of FU as a neoadjuvant for ALA-based PDT on mouse models of squamous cell carcinoma [50]. Pretreatment with FU for 3 days followed by ALA for 4 h led to large tumor-selective enhancement in protoporphyrin (PPIX) levels, increasing cell death upon illumination. In the study mentioned, upregulation of coproporphyrinogen oxidase and downregulation of ferrochelatase, which are key enzymes in the heme biosynthetic pathway, were identified to contribute to the improved therapeutic response on the combination of FU-ALA-PDT treatment. Additionally, a six-fold induction of p53 in FU pretreated tumors was reported.

In a similar manner, a combination FU and ALA using a mouse model of actinic keratosis showed that such binary therapy induced a long-term anti-tumor immune response and may synergize cytotoxic effects in cancer cells to provide better treatment efficacy in the management of actinic keratosis in the dermatology clinic [51].

It is entirely conceivable that our results with FU, presented here, follow similar mechanisms that come into play when FU was combined with our synthetic CDBTN PS. The decrease in cell proliferation observed is due to FU being known to cause inhibition of thymidylate synthase and production of metabolites incorporated into DNA to arrest tumor cellular growth. Exposure to FU leads to incorporation of fluorodeoxyuridine triphosphate (FdUTP) into DNA, and fluorouridine triphosphate into RNA, initiating DNA- and RNA-damage responses, respectively, thus causing p53 activation to control tumor cell cycle progression [52,53].

#### 3.1.4. Cell Death Pathways in Photodynamic Chemotherapy

The three conventional pillars of cell death pathways are apoptosis, necrosis, and autophagy. Apoptosis is programmed cell death characterized by nuclear fragmentation and the presence of apoptotic bodies [54,55]. Necrosis is unregulated cell digestion caused by external factors manifested as cell swelling and inflammatory response leading to premature cell death [56]. Autophagy or self-devouring is a natural degradation of cells to sequester unwanted or dysfunctional cellular components via a lysosomal-dependent regulated pathway, and characterized by the presence of autophagosomes, referred to as double-membrane vesicles containing degradation products that eventually fuse with lysosomes [57].

In recent years, the emergence of several non-conventional modalities that PDT-exposed cells go through has triggered interest in understanding the mechanism of PDT action. These observed non-conventional cell death machineries include mitotic catastrophe, paraptosis, and necroptosis, among others [41].

Mitotic catastrophe (MC) is a cell death pathway activated by aberrations in mitosis leading to chromosome disaggregation and cell division failure. It is considered a regulated oncologic suppression that obstructs cell survival due to extensive DNA damage and collapse of mitotic checkpoints. Characteristic morphological hallmarks of MC include specific nuclear changes such as multinucleation, macronucleation associated with chromosomal mis-aggregation, and micronucleation related to lagging or acentric chromosomes [58]. MC triggers a slow cell death response that culminates in morphological features closely resembling apoptotic or necrotic cell death or an irreversible senescent cell cycle arrest. PTX, being a member of the taxane family of chemotherapeutic agents, is known to directly affect DNA integrity or disrupt mitotic spindles, thereby impeding microtubule dynamics causing induction of MC [59].

A previous study conducted using α,β,χ,δ-porphyrin-tetrakis (1-methylpyridinium-4-yl) p-toluenesulfonate porphyrin (TMPyP) at 2.5 μM and a total light dose irradiation of 1 J/cm^2^ (20 s) was described as the first evidence of TMPyP-PDT induced microtubule disorganization of interphase cells, abnormal mitosis and formation of rounded cells with partial loss of adherence, with mitotic catastrophe on display [60]. The viability of PDT treated HeLa (human cervix adenocarcinoma) and G361 (human melanoma) cells in that particular in vitro study gradually decreased depending on post-PDT time.

Paraptosis is another nonconventional cell death mechanism characterized by cytoplasmic vacuolarization, and mitochondrial and/or endoplasmic reticulum (ER) swelling [61]. Contrary to apoptosis, paraptosis does not exhibit chromatin condensation and cell fragmentation. These morphological paraptotic features have been observed in PDT damage photoinduced by hypericin and benzoporphyrin derivatives (Verteporpfin) in human ovarian carcinoma cells (OVCAR-5) in which single-layered vacuoles were formed rather than double-layered ones, typically observed in autophagic cells [62].

Necroptosis is a regulated non-apoptotic form of inflammatory programmed cell death pathway closely resembling necrosis, often typified by cell swelling, moderate chromatin condensation, and rapid plasma membrane permeabilization with concomitant release of cellular debris into the extracellular space [63,64]. Nuclear fragmentation, internucleosomal DNA cleavage and caspase activation usually discerned in apoptosis are not present in necroptosis. Necroptotic cells do not exhibit shrinkage, but swelling and detachment accompanied by formation of nanoscale pores of more than 200 nm around the membrane are evident. Gradual reduction in cellular elasticity is also detected, hence cytoskeletal structure remains intact at the early stage of the necroptotic process [65].

Fluorescence and transmission electron microscopic techniques (Figure 7 and Figure 8) were used to visualize and examine the cell morphological features before and after photosensitization. Fluorescence and TEM images in our study seem to suggest that our synthesized CDBTN alone or in combination with PTX, CisPt, or FU follow apoptosis or autophagy, or some combination of these cell destruction mechanisms. The collected TEM cell morphology images do not seem to support other pathways such as paraptosis nor necroptosis as cellular swelling was not evident. Specific cell morphological characteristics can identify the mechanism of cell death, which can be exploited to understand the therapeutic outcome.

### 3.2. In Vivo Chemophotodynamic Treatment

The use of the hydra as a model organism for PDT application in this study is novel. Hydra is considered as one of the oldest animals in evolutionary terms with naturally occurring tumors as characterized by differentiation arrest and uncontrolled accumulation of female germline precursor cells. Previous studies indicated that a resident bacterium *Pseudomonas* and an environmental spirochete *Turneriella* induce tumorigenesis which significantly affected tissue homeostasis and healthy well-being of *Hydra oligactis* as the host organism [66,67]. Hydra as the model organism in our study was not infected with a bacteria to generate a tumor. This is the limitation of our study using the hydra as a model organism. However, we demonstrated that hydra can be used to study phototoxicity of PSs due to their low cost, ease of use, accessibility, and no strict regulation nor compliance that needs to be followed. The utility of hydra as a potential model organism for in vivo anti-cancer drug testing could open up a new and revolutionary avenue for future research due to its cost-effectiveness compared to mouse models.

## 4. Materials and Methods

### 4.1. Chemical Synthesis 

*General.* Desthiobiotin, solvents and reagents were purchased mainly from Sigma (Sigma Aldrich Chemical Co., St. Louis, MO, USA). All air- and moisture-sensitive reactions were performed in anhydrous solvents under nitrogen atmosphere. Chromatographic purifications were performed in normal phase preparative Analtech (Fisher, Waltham, MA, USA) TLC (thin-layer chromatography) plate. Reactions were monitored using polyester-backed normal phase analytical silica gel 60 F_254_ precoated 200 μm TLC plates (Thermo Fisher Scientific, Waltham, MA, USA) and detected with UV light (λ = 254 nm). NMR spectra were acquired with an NMR spectrometer (Bruker Corporation, Billerica, MA USA) Avance version (400 MHz for ^1^H and 100 MHz for ^13^C). Chemical shifts are reported in *δ* ppm referenced according to the deuterated solvents used as internal standards: CDCl_3_ 7.24 ppm (^1^H) and 77.23 ppm (^13^C). High resolution mass spectra were obtained on a micrOTOF-II ESI mass spectrometer (Bruker Corporation, Billerica, MA, USA). All compounds synthesized were isolated and purified in ≥95% purity as confirmed by ^1^H, ^13^C, 2D COSY (Correlated Spectroscopy), DEPT 135 (Distortion-less Enhancement by Polarization Transfer), and HSQC (Heteronuclear Single Quantum Correlation) NMR spectra. Sample purity was also checked using HPLC (high performance liquid chromatography) Ultimate 3000 (Thermo Fisher Scientific, Waltham, MA, USA) equipped with a diode-array four-channel variable UV-visible detector, an autosampler and a fraction collector using a reverse-phase column (C-18, 4.6 × 50 mm, 3.5 μm) in isocratic mobile phase (100% MeOH) visualizing at λ = 405 and 665 nm with a flow rate of 1 mL/min.

*Synthesis of 13^1^-Hexamethylenediaminyl-desthiobiotinylchlorin e_6_ dimethyl ester, CDBTN, 3.* Starting with 38.4 mg (0.179 mmol) of desthiobiotin, 54.8 mg (0.198 mmol) of DMTMM [4-(4,6-dimethoxy-1,3,5-triazin-2-yl)-4-methyl-morpholinium chloride], and 8.5 mL of dry chloroform were added to the round bottom flask containing 68 mg (0.094 mmol) of MePheo-hexyl-NH_2_ 2. The flask was covered with a septum and allowed to stir under a nitrogen balloon for 3 days. The reaction was checked with a TLC plate, using 5% MeOH in DCM (dichloromethane, CH_2_Cl_2_). To purify the product, a preparative silica plate was used in 5% MeOH in DCM for 40 min. The large dark band was scraped off and dissolved in the leftover 5% MeOH in DCM, and the silica was filtered out. This solution was evaporated using a rotary evaporator to obtain 76.3 mg (0.083 mmol) of the target compound CDBTN 3 (88.2% yield). Product purity was determined using reverse-phase HPLC with MeOH as the mobile phase. UV-Vis (CH_2_Cl_2_): *λ*_max_ (*ε*/M^−1^cm^−1^) 662 (37,720), 606 (3307), 528 (2893), 502 (10,413), 402 (121,853); ^1^H NMR (CDCl_3_, 400 MHz): δ 9.62 (s, 1H, 10-meso H), 9.57 (s, 1H, 5-meso H), 8.76 (s, 1H, 20-meso H), 8.01–7.93 (dd, *J* = 17.91, 11.64 Hz, 1H, 3^1^C*H* = CH_2_), 7.21 (br s, 1H, -N*H*CH_2_(CH_2_)_4_CH_2_NH-), 6.29–6.22 (d, *J* = 17.64 and 1.06 Hz, 1H, trans 3^2^CH = C*H*_2_), 6.07–6.02 (d, *J* = 11.40 and 1.04 Hz, 1H, cis 3^2^CH = C*H*_2_), 5.86 (br s, 1H, -NHCH_2_(CH_2_)_4_CH_2_N*H*-), 5.54–5.25 (br m, 2H, 15^1^C*H*_2_), 4.81 (br s, 1H, DBTN ring N-H), 4.41 (q, J = 7.08 Hz, 1H, 18-H), 4.26 (br m, 1H, 17-H), 4.15 (br s, 1H, DBTN ring N-H), 3.69 (s and overlapping m, 3H, 15^3^CO_2_C*H*_3_, and 2H, 8^1^-C*H*_2_), 3.55 (s, 3H, 17^4^CO_2_C*H*_3_), 3.46 (s, 3H, 12^1^-C*H*_3_, and 2H, -NHC*H*_2_(CH_2_)_4_CH_2_NH-), 3.40 (s, 3H, 2^1^-C*H*_3_,) 3.22 (s, 3H, 7^1^-C*H*_3_), 3.06 (br q, 2H, -NHCH_2_(CH_2_)_4_C*H*_2_NH-), 2.97 (br m, 1H, DBTN ring C*H*_B_), 2.78 (br m, 1H, DBTN ring C*H*_C_), 2.48–2.14 (br m, 2H, 17^2^C*H*_2_), 2.14 (br m, 2H, 17^1^-C*H*_2_), 1.76 (br t, 2H, DBTN-C*H*_H2_-CONH-), 1.64 (br overlapping m, 3H, J = 7.08 Hz, 18^1^-C*H*_3_, and 3H, 8^2^-C*H*_3_), 1.37–1.29 (br m, 8H, -NHCH_2_(C*H*_2_)_4_CH_2_NH-), 1.19 (br m, 2H, O = C-CH_2_C*H*_G2_CH_2_CH_2_CH_2_-DBTN-ring), 0.79 (overlapping m, O = C-(CH_2_)_2_C*H*_F2_C*H*_E2_C*H*_D2_-DBTN-ring), 0.46 (d, 3H, C*H*_A3_-DBTN ring), −1.89—−2.03 (br s, 2H, ring NH); ^13^C-NMR (CDCl_3_, 100 MHz): δ 174.2 (DBTN-L *C* = O), 173.5 (13^1^-*C*ONH), 173.0 (side chain DBTN-K *C* = O), 169.3 (15^2^-*C*O_2_CH_3_), 169.1 (17^4^-*C*O_2_CH_3_), 167.3 (13), 163.1 (1), 160.0 (4), 152.2 (6), 144.4 (8), 139.0 (9), 135.9 (11), 135.6 (2), 135.5 (12), 134.8 (14), 134.6 (15), 130.3 (3^1^), 129.4 (7), 129.1 (16), 121.9 (3^2^), 102.7 (19), 101.1 (10), 98.8 (5), 94.0 (20), 55.4 (*C*_C_H, DBTN ring), 53.2 (17), 52.8 (15^3^CO_2_*C*H_3_), 51.6 (17^4^CO_2_*C*H_3_), 50.8 (*C*_B_NH- DBTN ring), 49.2 (18), 40.3 (-NH-*C_a_*H_2_(CH_2_)_4_CH_2_-NHCO-DBTN), 39.0 (-NH-CH_2_(CH_2_)_4_*C_f_*H_2_-NHCO-DBTN), 37.7 (15^1^), 35.8 (-NHCO-*C*_H_H_2_-, DBTN alkyl chain), 31.1 (17^2^), 29.7 (17^1^), 29.4 (*C*_F_H_2_, DBTN alkyl chain), 29.3 (*C*_D_H_2_, DBTN alkyl chain), 28.9–28.3 (-NH-CH_2_*C*_b_H_2_(CH_2_)_2_*C*_e_H_2_CH_2_NHCO-DBTN ring), 26.5–26.3, (-NH-(CH_2_)_2_*C*_c_H_2_*C*_d_H_2_(CH_2_)_2_NH-CO-DBTN ring), 25.3 (*C*_E_H_2_, DBTN alkyl chain), 25.0 (*C*_G_H_2_, DBTN alkyl chain), 23.2 (8^2^), 19.7 (8^1^), 17.6 (18^1^), 15.1 (*C*_A_H_3_-DBTN ring), 12.2 (12^1^), 12.0 (2^1^), 11.3 (7^1^); HPLC (100% MeOH), t_R_: 1.480 min (96.9%); HRMS (ESI) *m/z* 919.5446 [M^+^], calcd for C_52_H_70_N_8_O_7_ 919.5211.

*Synthesis of Zn(II)-13^1^-Hexamethylenediaminyl-desthiobiotinoylchlorin e_6_ dimethyl ester, ZnCDBTN, 4*. In a round bottom flask, CDBTN 3 (14.7 mg, 0.016 mmol) was dissolved in 7.5 mL of saturated Zn(OAc)_2_ in MeOH and 15 mL of DCM. The solution was refluxed for 30 min and then allowed to cool. Completion of metallation was monitored using the UV/Vis spectrophotometer. Once the reaction was completed, the solution was rinsed once with H_2_O, twice with NaHCO_3_ (aqueous saturated solution), and then once with NaCl (aqueous saturated solution). The solution was then dried over Na_2_SO_4_. To purify the product, a preparative silica plate was used using 10% MeOH in DCM as the mobile phase for 40 min. The large dark band was scraped off and dissolved in 10% MeOH in DCM, and the silica was filtered out. The solvent was removed using a rotary evaporator to yield 13.9 mg (0.014 mmol) of the target ZnCDBTN 4 (88.5% yield). Purity of the Zn complex was checked using reverse-phase HPLC with MeOH as the mobile phase. UV-vis (CH_2_Cl_2_): *λ*_max_ (*ε*/M^−1^cm^−1^) 638 (56,733), 594 (10,840), 516 (7427), 416 (154,720); ^1^H NMR (CDCl_3_, 400 MHz): δ 9.37 (br s, 2H, 10- and 5-meso H), 8.41 (s, 1H, 20-meso H), 7.92 (very br m, 1H, 3^1^C*H* = CH_2_), 6.92 (br s, 1H, -N*H*CH_2_(CH_2_)_4_CH_2_NH-), 6.22 (br s, 1H, trans 3^2^CH = C*H*_2_), 6.06–6.00 (br m, 1H, cis 3^2^CH = C*H*_2_), 5.85 (br m, 1H, -NHCH_2_(CH_2_)_4_CH_2_N*H*-), 5.35–5.27 (br m, 2H, 15^1^C*H*_2_), 4.92–4.87 (br m, 1H, DBTN ring N-H), 4.20–4.09 (br m, 1H, 18-H; 1H, DBTN ring N-H; 1H, 17-H), 3.70 (br s and overlapping m, 3H, 15^3^CO_2_C*H*_3_, and 2H, 8^1^-C*H*_2_), 3.36 (s, 3H, 17^4^CO_2_C*H*_3_, and 2H, -NHC*H*_2_(CH_2_)_4_CH_2_NH-), 3.26–3.21 (br overlapping m, 3H, 12^1^-C*H*_3_; 3H, 2^1^-C*H*_3_; 3H, 7^1^-C*H*_3;_ 2H, -NHCH_2_(CH_2_)_4_C*H*_2_NH-; 2H, DBTN ring C*H*_B_; 2H, DBTN ring C*H*_C_), 2.43–2.13 (br m, 2H, 17^2^C*H*_2_), 2.26 (br m, 2H, 17^1^-C*H*_2_), 2.00 (br s, 3H, 18^1^-C*H*_3_), 1.72 (br s, 2H, DBTN-C*H*_H2_-CONH-), 1.68–1.58 (br m, 3H, 8^2^-C*H*_3_), 1.12–1.00 (br m, 8H, -NHCH_2_(C*H*_2_)_4_CH_2_NH-), 0.74 (br m, 2H, O = C-CH_2_C*H*_G2_CH_2_CH_2_CH_2_-DBTN-ring), 0.52 (overlapping m, O = C-(CH_2_)_2_C*H*_F2_C*H*_E2_C*H*_D2_-DBTN-ring), 0.01 (d, 3H, C_A_*H*_3_-DBTN ring); ^13^C-NMR (CDCl_3_, 100 MHz): δ 174.8 (DBTN-L *C* = O), 173.9 (13^1^-*C*ONH), 173.2 (side chain DBTN-K *C* = O), 172.0 (15^2^-*C*O_2_CH_3_), 171.2 (17^4^-*C*O_2_CH_3_), 165.4 (13), 160.2 (1), 157.3 (4), 152.9 (6), 146.8 (8), 141.8 (9), 133.6 (11), 133.1 (2), 132.8 (12), 130.8 (14), 130.0 (15), 126.2 (3^1^), 125.5 (7), 124.7 (16), 119.5 (3^2^), 105.6 (19), 104.4 (10), 103.2 (5), 90.3 (20), 54.9 (*C*_C_H, DBTN ring), 52.9 (17), 52.0 (15^3^CO_2_*C*H_3_), 51.4 (17^4^CO_2_*C*H_3_), 49.8 (*C*_B_NH- DBTN ring), 47.6 (18), 38.3 (-NH-*C_a_*H_2_(CH_2_)_4_CH_2_-NHCO-DBTN), 38.2 (-NH-CH_2_(CH_2_)_4_*C_f_*H_2_-NHCO-DBTN), 37.7 (15^1^), 36.8 (-NHCO-*C*_H_H_2_-, DBTN alkyl chain), 30.2 (17^2^), 29.7 (17^1^), 29.5 (*C*_F_H_2_, DBTN alkyl chain), 28.8 (*C*_D_H_2_, DBTN alkyl chain), 28.7–28.2 (-NH-CH_2_*C*_b_H_2_(CH_2_)_2_*C*_e_H_2_CH_2_NHCO-DBTN ring), 25.7–25.1 (-NH-(CH_2_)_2_*C*_c_H_2_*C*_d_H_2_(CH_2_)_2_NHCO-DBTN ring), 24.5 (*C*_E_H_2_, DBTN alkyl chain), 24.4 (*C*_G_H_2_, DBTN alkyl chain), 22.8 (8^2^), 19.5 (8^1^), 17.9 (18^1^), 14.5 (*C*_A_H_3_-DBTN ring), 12.4 (12^1^), 12.1 (2^1^), 11.4 (7^1^); HPLC (100% MeOH), t_R_: 1.403 min (90.3%); HRMS (MALDI-TOF) *m/z* 981.4566 [M^+^], calcd for C_52_H_68_N_8_O_7_Zn 981.4575.

*Synthesis of 13^1^-Hexamethylenediaminyl-desthiobiotinoylchlorin e_6_ dimethyl ester indium (III) chloride, InCDBTN-Cl, 5*. In a round bottom flask, 50 mg (0.054 mmol) of CDBTN 3, 425 mg of NaOAc and 250 mg (1.136 mmol) of InCl_3_, and 5 mL of HOAc were added. The solution was refluxed for 12 h and then allowed to cool. The solution was slowly added to about 70 mL of water in a beaker. A saturated aqueous solution of NaHCO_3_ was added to the solution slowly and then the resulting mixture was transferred into a separatory funnel. Aqueous saturated solutions of NaCl and DCM were added to the solution, and the DCM layer was extracted. The solution was re-extracted using ethyl acetate. The DCM and ethyl acetate layers were combined. This mixture was dried using Na_2_SO_4_ and then evaporated using a rotary evaporator to obtain 17 mg (0.016 mmol) of the InCDBTN-Cl target compound 5 (29.3% yield). The InCDBTN-Cl product was checked for purity using reverse-phase HPLC with MeOH as the mobile phase. UV-vis (CH_2_Cl_2_): *λ*_max_ (*ε*/M^−1^cm^−1^) 636 (41,467), 596 (9800), 524 (6693), 418 (168,133); ^1^H NMR (CDCl_3_, 400 MHz): δ 9.62 (s, 1H, 10-meso H), 9.58 (s, 1H, 5-meso H), 8.62 (s, 1H, 20-meso H), 7.97–7.89 (dd, *J* = 17.91, 11.64 Hz, 1H, 3^1^C*H* = CH_2_), 6.92 (br s, 1H, -N*H*CH_2_(CH_2_)_4_CH_2_NH-), 6.19–6.15 (d, *J* = 17.64 and 1.06 Hz, 1H, trans 3^2^CH = C*H*_2_), 6.06–6.03 (d, *J* = 11.40 and 1.04 Hz, 1H, cis 3^2^CH = C*H*_2_), 6.03 (br s, 1H, -NHCH_2_(CH_2_)_4_CH_2_N*H*-), 5.44–5.40 (br m, 2H, 15^1^C*H*_2_), 5.09 (br s, 1H, DBTN ring N-H), 4.43- 4.38 (q, J = 7.08 Hz, 1H, 18-H, and 1H, DBTN ring N-H), 4.31 (br m, 1H, 17-H), 3.72 (br s and overlapping m, 3H, 15^3^CO_2_C*H*_3_, and 2H, 8^1^-C*H*_2_), 3.58 (br overlapping m, 3H, 17^4^CO_2_C*H*_3_, and 2H, -NHC*H*_2_(CH_2_)_4_CH_2_NH-), 3.45 (br t, 2H, -NHCH_2_(CH_2_)_4_C*H*_2_NH-), 3.41 (br s, 3H, 12^1^-C*H*_3_), 3.30 (s, 3H, 2^1^-C*H*_3_,) 3.28(s, 3H, 7^1^-C*H*_3_,), 3.19 (br m, 2H, DBTN ring C*H*_B_ and C*H*_C_), 2.59–2.16 (br m, 2H, 17^2^C*H*_2_), 2.25 (br s, 2H, 17^1^-C*H*_2_), 2.05 (br t, J = 7.08 Hz, 3H, 18^1^-C*H*_3_), 1.76 (br t, 2H, DBTN-C*H*_H2_-CONH-), 1.65 (br t, J = 7.08 Hz, 3H, 8^2^-C*H*_3_), 1.51–1.47 (br m, 8H, -NHCH_2_(C*H*_2_)_4_CH_2_NH-), 1.41 (br m, 2H, O = C-CH_2_C*H*_G2_CH_2_CH_2_CH_2_-DBTN-ring), 1.21 (overlapping m, O = C-(CH_2_)_2_C*H*_F2_C*H*_E2_C*H*_D2_-DBTN-ring), 0.80 (d, 3H, C_A_*H*_3_-DBTN ring); ^13^C-NMR (CDCl_3_, 100 MHz): δ 175.7 (DBTN-L *C* = O), 174.9 (13^1^-*C*ONH), 174.6 (side chain DBTN-K *C* = O), 173.7 (15^2^-*C*O_2_CH_3_), 173.3 (17^4^-*C*O_2_CH_3_), 169.8 (13), 163.8 (1), 161.2 (4), 153.1 (6), 144.2 (8), 140.8 (9), 135.4 (11), 135.0 (2), 134.8 (12), 133.9 (14), 133.6 (15), 129.6 (3^1^), 128.8 (7), 128.3 (16), 122.1 (3^2^), 104.2 (19), 103.0 (10), 102.0 (5), 93.5 (20), 56.0 (*C*_C_H, DBTN ring), 53.4 (17), 52.3 (15^3^CO_2_*C*H_3_), 51.8 (17^4^CO_2_*C*H_3_), 51.3 (*C*_B_NH- DBTN ring), 50.2 (18), 40.3 (-NH-*C_a_*H_2_(CH_2_)_4_CH_2_-NHCO-DBTN), 39.2 (-NH-CH_2_(CH_2_)_4_*C_f_*H_2_-NHCO-DBTN), 38.0 (15^1^), 36.0 (-NHCO-*C*_H_H_2_-, DBTN alkyl chain), 30.3 (17^2^), 29.7 (17^1^), 29.4 (*C*_F_H_2_, DBTN alkyl chain), 29.4 (*C*_D_H_2_, DBTN alkyl chain), 29.1–28.5 (-NH-CH_2_*C*_b_H_2_(CH_2_)_2_*C*_e_H_2_CH_2_NHCO-DBTN ring), 26.6–26.3 (-NH-(CH_2_)_2_*C*_c_H_2_*C*_d_H_2_(CH_2_)_2_NHCO-DBTN ring), 25.6 (*C*_E_H_2_, DBTN alkyl chain), 25.3 (*C*_G_H_2_, DBTN alkyl chain), 20.8 (8^2^), 19.5 (8^1^), 17.4 (18^1^), 15.3 (*C*_A_H_3_-DBTN ring), 12.2 (12^1^), 12.1 (2^1^), 11.2 (7^1^); HPLC (100% MeOH), t_R_: 1.213 min (97.2%); HRMS (MALDI-TOF) *m/z* 1031.4245 [M^+^-Cl], calcd for C_52_H_68_ClInN_8_O_7_ 1031.4283 [-Cl]. 

### 4.2. In Vitro Cytotoxicity Assay

*General*. A human mammary epithelial carcinoma cell line purchased from the American Type Culture Collection BT-549 (ATCC HTB-122) was cultured according to the ATCC protocol. Briefly, BT-549 cells were grown in RPMI 1640 containing 0.023 IU/mL insulin and supplemented with 10% fetal bovine serum (FBS). Cells were grown to 80–90% confluence in 75 cm^2^ culture flasks (Corning) for 4–5 days in a humidified incubator (Fisher Scientific Isotemp) with 5% CO_2_ at 37 °C. During the incubation period, growth media was changed once with fresh pre-warmed media (pH 7.2). To harvest the cells, old growth media was aspirated out and 3 mL 0.25% trypsin solution (ThermoSci Hyclone) was added. The cells were incubated for 5 min and the cell pellet after centrifugation was resuspended in 3 mL media, broken up gently, then 1 mL of suspended cells was transferred into a new T75 cell culture flask containing pre-warmed media (20 mL) for further culturing.

*Cell viability assay.* Cells were grown to confluence in a 96-well plate (5 × 10^4^ cells/well) and treated for 24 h with photosensitizers of varying concentrations (ranging from 10 nM to 100 nM or in combination with chemotherapeutic drugs (50 nM PTX, 25 μM CisPt or 25 μM FU) in growth media from a stock solution of 10 mM in DMSO (dimethyl sulfoxide, Fisher). After 24 h treatment, old growth media containing the PS or compounds were aspirated out and replaced with fresh media. Plates were then positioned below a non-coherent LumaCare LC-122 650 nm light source for 1, 2, and 5 min at an energy fluence rate of 16 mW/cm^2^ (measured using a Newport optical power meter Model 840). Unirradiated cells served as control samples. The following day, cells were washed with pre-warmed PBS, and MTT (3-[4,5-dimethyl-thiazol-2-yl]-2.5-diphenyltetrazolium bromide, Sigma, 0.3 mg/mL) in PBS was added to each well [39,40]. Samples were allowed to incubate for an additional 2 h, after which dark blue crystals formed. DMSO was added to each well and plates were shaken at room temperature for 1 h to dissolve the purplish-blue formazan crystals. Absorbance values at 570 nm were measured on a BioRad 550 (BioRad, Hercules, CA, USA) microplate reader. Cell survival was calculated based on the absorbance of the untreated cells alone (as control) and was directly proportional to the number of viable cells in culture. Results were reported as the mean ± SD of triplicate measurements.

*Fluorescence microscopy*. Cells (1 mL aliquots) obtained from a diluted cell suspension were seeded into each well (1.7 cm^2^, 5 × 10^3^ cells/well) of a 4-well culture slide (BD Biosciences, San Jose, CA, USA) and grown to confluence in 5% CO_2_ at 37 °C for 3–4 days for attachment to the substratum. After aspirating the old growth media, 1 mL of the compound or photosensitizer (with varying concentrations or in combination with chemotherapeutic drugs (PTX, CisPt or FU) in fresh pre-warmed media at 37 °C was added to each well. After compound treatment for 24 h, cells were washed twice with 1 mL fresh growth media, and then irradiated with light using LumaCare LC-122 as described above. Cells were stained in the dark with Hoechst 33258 (Molecular Probes, Thermo Fisher Scientific, Waltham, MA, USA) in pre-warmed media for 10 min at 37 °C, washed twice with filtered PBS, then fixed with filtered paraformaldehyde for 15 min in the incubator. After thorough liquid aspiration, the wells were removed and allowed to air dry in the dark for 1 h. Slides were protected with coverslips, the edges of which were sealed using a clear fast-drying nail polish and allowed to dry at room temperature in the dark for 30 min. Images were recorded using fluorescence microscopy (DAPI for Hoechst 350–390 nm excitation and 460–490 nm emission filters) using an upright fluorescence microscope Nikon Optiphot-2 (Yashima Optical Co., Tokyo, Japan) with Retiga imaging 2000R (20× and 40×) and an image processing Nikon NIS-Elements V4.0 Qimaging software.

*Transmission Electron Microscopy*. Cells were cultured to confluence in a petri dish (50 cm in diameter), treated for 24 h with 500 nM of PS or with chemotherapeutic drugs (PTX, CisPt or FU), or in combination, then irradiated for 2 min as above. After photosensitization 24 h later, cells were scraped gently in the dark, fixed in 2.5% glutaraldehyde in 0.1 M sodium cacodylate buffer, then post-fixed with 1% osmium tetroxide (containing 0.8% ferricyanide), treated with 2% aqueous uranyl acetate, and subsequently dehydrated in gradient concentrations (50–100%) of varying ethanol/water mixtures. The resulting pellets were embedded in resin and subsequently cut with an ultramicrotome to a 70 nm thickness, then viewed using a Tecnai T20 transmission electron microscope.

*Dose–Effect and Drug Combination Analysis.* Drug combination index was calculated based on the Chou–Talalay method Version 1.0 available online as freeware (https://www.combosyn.com, accessed on 8 January 2025). The program provided quantitation of synergism and antagonism in drug combinations. The combination index (CI) was then determined based on the algorithm using experimental data derived from the cell cytotoxicity assay of PSs alone and PS combined with chemotherapeutic agents. Specific concentrations used from single doses of PS and chemotherapeutic agent, and combinations are entered. The CI index calculated was matched using the Chou-MartinCompuSyn User’s Guide (Copyright 2005) Table 4.1 (p. 34) (https://combosyn.com/Content2012/Users%20Guide.pdf, accessed on 8 January 2025) with the CI indices ranging from <0.1 to 1.00 to >10 being described as very strongly synergistic to additive to very strongly antagonistic, respectively.

*Statistical Analysis.* Statistical analysis for the effect of the combination treatment was conducted independently with separate and appropriate controls for the different combination treatment of PS and chemotherapeutic drugs. Results were represented as mean ± standard error. Replicate measurements were performed three times with assays run in triplicate. Statistical significance defined as *p* (probability) value less than 0.05 was evaluated using the two-tailed *t*-test (paired samples for means) available in the Data Analysis toolbox in Excel.

### 4.3. In Vivo Cytotoxicity Assay

*General*. Green hydra (*Hydra viridissima*) were purchased from Flinn Scientific and were grown in petri dishes. Hydra culture was maintained at room temperature and the media consisted of mixing 4 mL of Stock Solution 1 (42.18 g CaCl_2_.2H_2_O in 1 L of milliQ H_2_O, sterile filtered) and 40 mL of Stock Solution 2 (8.116 g MgSO_4_.7H_2_O, 4.238 g NaHCO_3_, and 1.0985 g K_2_CO_3_ in 1 L of milliQ H_2_O), then diluted to 4 L of milliQ H_2_O [13]. The hydras were fed with brine shrimps hatched from the eggs of *Artemia nauplii* (Brine Shrimp Direct) in the presence of light. The brine shrimp media consisted of 37.5 g of non-iodized Morton salt, 0.5 g sodium bicarbonate, 1.5 L milliQ H_2_O and three scoops of *Artemia* eggs. After 2 days, the freshly-hatched shrimps were used to feed the hydra. Each hydra animal can consume 3–4 shrimps (in 30–40 min mealtime), and fed once a week. To maintain hydra health and prevent microbial contamination, the media was replaced with fresh hydra media after 2 h of feeding (to ensure that no left-over brine shrimp nor any other debris is present), and once or twice during the week.

*Hydra photodynamic efficacy assay.* Hydras were grown in a 6-well plate. One animal occupied one well, and they were treated for 24 h with 50 nM CDBTN dissolved in the hydra media from a stock solution of 10 mM in DMSO. After 24 h treatment, old hydra media containing the PS were aspirated out and replaced with fresh hydra media. Plates were then positioned below a non-coherent LumaCare LC-122 650 nm light source and irradiated for 1, 2, and 5 min at an energy fluence rate of 16 mW/cm^2^ (measured using a Newport optical power meter Model 840). Unirradiated hydras served as control samples. After 24 h, the hydras were viewed under an Olympus CK2 (Olympus Optical Co., Tokyo, Japan) inverted microscope (10× objective) equipped with an Olympus OM-m4/3 lens mount adapter that mounted a Panasonic Lumix DMC-GH1 camera to capture images and examine their morphology. Three trials corresponding to three hydras for each condition were performed.

## 5. Conclusions

The study presented here, as well as in numerous other research investigations, demonstrates that the integration of chemotherapy and photodynamic or light therapy have better anti-tumor efficacy than monotherapies, and may provide an option in lessening the devastating toxic side effects associated with chemotherapy and boost therapeutic efficiency associated with a single therapeutic approach. Our synthesized CDBTN with non-existent dark cytotoxicity can be a promising photosensitizer for PDT applications and combined PDT chemotherapy TNBC treatment. Identifying the mode of cellular mechanistic destruction triggered by PDT is important in understanding and optimizing treatment outcomes. The use of cost-effective animal models is necessary to translate results in vitro into clinical studies. Future research investigation for pre-clinical studies could involve photodynamic efficiency of the synthesized photosensitizers in widely accepted animal models of TNBC.

Since its inception in the 1970s, challenges facing the widespread clinical application of PDT have been and still are daunting, but PDT is gradually becoming an attractive technology for cancer treatment. PDT has been proven to have outstanding performance in cancer therapy, but disadvantages persist, including limited light penetration depth, poor tumor selectivity, and oxygen dependence, which largely restrict its therapeutic efficiency for deeply seated solid tumors. Several strategies have been explored to overcome these barriers, such as designing new photosensitizers with higher photodynamic conversion efficiency, alleviating tumor hypoxia to generate reactive oxygen species (ROS), and applying PDT-based combination strategies. The advent of nanotechnology in multifunctional platforms has shown some promise in improving PDT outcomes in the laboratory setting. Continued collective research efforts are underway in this area and more studies are needed to prove its efficacy for clinical applications.

The PDT technique is a viable option for TNBC patients due to its marginal toxicity, repeatability, immunological effects and minimally invasive procedure. PDT can also provide breast preservation as an additional advantage for women patients. PDT with other complementary therapeutic routes such as chemotherapy, photothermal therapy, immunotherapy, gas therapy, and calorie restriction are being combined to resolve pending challenges and problems of PDT as a monotherapy. Among numerous anti-cancer modalities, PDT will continue to attract attention as a minimally invasive therapeutic approach, and may have applications as adjuvant and/or neoadjuvant treatments to conventional treatment such as radiotherapy or chemotherapy.

## Figures and Tables

**Table 1:** Average cell inhibition (%) of TNBC cells treated with CDBTN photosensitizer alone and with combined CDBTN and chemotherapeutic drugs for 24 h, followed by light irradiation (light dose of 0.96 J cm^−2^). Chemotherapeutic drugs: PTX (50 nM), CisPt (25 μM), and FU (25 μM).

[CDBTN] Concentration	CDBTN Only	PTX 50 nM	CisPt 25 μM	FU 25 μM
50 nM	34%	66%	41%	80%
75 nM	59%	72%	65%	93%
100 nM	87%	81%	93%	95%

**Table 2:** Combination Index (CI) values of binary therapy (CDBTN as the PS and chemotherapeutic drugs) and the corresponding synergism vs. additive vs. antagonism effects. CI = 1 (additive); CI < 1 (synergism); CI > 1 (antagonism). VSS (<0.1), very strong synergism; SS (0.1–0.3), strong synergism; S (0.3–0.7), synergism; MS (0.7–0.85), moderate synergism; StS (0.85–0.90), slight synergism; NA (0.9–1.10), nearly additive; StA (1.10–1.20), slight antagonism; MA (1.20–1.45), moderate antagonism; A (1.45–3.3), antagonism; SA (3.3–10), strong antagonism; VSA (>10), very strong antagonism. Concentrations: PSs = 50 and 75 nM; taxol = 50 nM; CisPt = 25 μM; FU = 25 μM.

CDBTN Concentration	Taxol 50 nM	CisPt 25 μM	FU 25 μM
50 nM	0.70 (S)	1.37 (MA)	0.55 (S)
75 nM	0.95 (NA)	1.20 (SA)	0.59 (S)

## Data Availability

Data are contained in this article.

## Abbreviations

PDT, photodynamic therapy; PS, photosensitizer; TNBC, triple-negative breast cancer; ER, estrogen receptor; PR, progesterone receptor; HER2, human epidermal growth factor 2 receptor; HER1, human epidermal growth factor 1 receptor; SMVT, sodium-dependent multivitamin transporter; DBTN, desthiobiotin; CDBTN, chlorin-desthiobiotin; InCDBTN–Cl, indium (III) chloride complex of CDBTN; ZnCDBTN, zinc (II) complex of CDBTN; Boc, *tert*-butoxy carbonyl; TFA, trifluoroacetic acid; NaCl, sodium chloride; NaHCO_3_, sodium bicarbonate; MeOH, methanol; CH_2_Cl_2_, dichloromethane or methylene chloride; NaOAc, sodium acetate; HOAc, acetic acid; Zn(OAc)_2_, zinc acetate; DMTMM, 4-(4,6-dimethoxy-1,3,5-triazin-2-yl)-4-methylmorpholinium chloride; DME, dimethyl ester; NMR, nuclear magnetic resonance; CDCl_3_, deuterated chloroform; HPLC, high performance liquid chromatography; HRMS, high resolution mass spectrometry; PTX, paclitaxel; CisPt, cisplatin; FU or 5-FU, fluorouracil or 5-fluorouracil; CI, combination index.
